# Development and validation of a nomogram to predict poor efficacy of imatinib in the treatment of newly diagnosed chronic phase chronic myeloid leukemia patients

**DOI:** 10.3389/fonc.2024.1418417

**Published:** 2024-06-24

**Authors:** Yuxin Li, Yilin Zhang, Jin Wang, Aili He, Wanggang Zhang, Xingmei Cao, Yinxia Chen, Jie Liu, Pengyu Zhang, Jianli Wang, Wanhong Zhao, Yun Yang, Xin Meng, Sheping Chen, Longjin Zhang, Ting Wang, Xugeng Wang, Xiaorong Ma

**Affiliations:** Department of Hematology, The Second Affiliated Hospital of Xi’an Jiaotong University, Xi’an, Shaanxi, China

**Keywords:** chronic phase chronic myeloid leukemia, imatinib, prognostic model, nomogram, Sokal score, Euro score, EUTOS score, ELTS score

## Abstract

**Background:**

Imatinib is the most widely used tyrosine kinase inhibitor (TKI) in patients with newly diagnosed chronic-phase chronic myeloid leukemia(CML-CP). However, failure to achieve optimal response after imatinib administration, and subsequent switch to second-generation TKI therapy results in poor efficacy and induces drug resistance. In the present study, we developed and validated a nomogram to predict the efficacy of imatinib in the treatment of patients newly diagnosed with CML-CP in order to help clinicians truly select patients who need 2^nd^ generation TKI during initial therapy and to supplement the risk score system.

**Methods:**

We retrospectively analyzed 156 patients newly diagnosed with CML-CP who met the inclusion criteria and were treated with imatinib at the Second Affiliated Hospital of Xi’an Jiao Tong University from January 2012 to June 2022. The patients were divided into a poor-response cohort (N = 60)and an optimal-response cohort (N = 43) based on whether they achieved major molecular remission (MMR) after 12 months of imatinib treatment. Using univariate and multivariate logistic regression analyses, we developed a chronic myeloid leukemia imatinib-poor treatment (CML-IMP) prognostic model using a nomogram considering characteristics like age, sex, HBG, splenic size, and ALP. The CML-IMP model was internally validated and compared with Sokal, Euro, EUTOS, and ELTS scores.

**Results:**

The area under the curve of the receiver operator characteristic curve (AUC)of 0.851 (95% CI 0.778–0.925) indicated satisfactory discriminatory ability of the nomogram. The calibration plot shows good consistency between the predicted and actual observations. The net reclassification index (NRI), continuous NRI value, and the integrated discrimination improvement (IDI) showed that the nomogram exhibited superior predictive performance compared to the Sokal, EUTOS, Euro, and ELTS scores (*P* < 0.05). In addition, the clinical decision curve analysis (DCA) showed that the nomogram was useful for clinical decision-making. In predicting treatment response, only Sokal and CML-IMP risk stratification can effectively predict the cumulative acquisition rates of CCyR, MMR, and DMR (*P*<0.05).

**Conclusion:**

We constructed a nomogram that can be effectively used to predict the efficacy of imatinib in patients with newly diagnosed CML-CP based on a single center, 10-year retrospective cohort study.

## 1 Introduction

Since the introduction of the tyrosine kinase inhibitor (TKI); imatinib, the prognosis of patients with chronic phase chronic myeloid leukemia(CML-CP) has greatly improved, with a 10-year overall survival (OS) of 80–90% (1, 2). The emergence of 2^nd^ generation TKI drugs (e.g., nilotinib and dasatinib) has enabled patients with CML to achieve faster and deeper molecular responses (3–6). Imatinib mesylate is the most widely used drug in patients with chronic-phase CML owing to its safety, efficacy, and pharmacoeconomic advantages. Data show that patients who were initiated with imatinib therapy but failed to meet certain response landmarks such as those proposed in the 2020 European Leukemia Net (ELN) criteria and who were switched to a 2^nd^ or 3^rd^ generation TKI had poor outcomes and easily developed drug resistance, which led to a poor long-term survival (7–11). Therefore, it is extremely important to help clinicians select patients who need 2^nd^ generation TKI treatment as an initial therapy.

Current prognostic scoring systems such as the Sokal, Euro, EUTOS, and ELTS are widely used in clinical practice but they have limitations (12–15). The Sokal and Euro scoring systems were established before the advent of imatinib, and the OS of patients was considered the endpoint of the study. After the advent of imatinib, the survival rate was greatly improved, and the pursuit of survival was quicker molecular responses and higher rates of major molecular response (MMR, ≤ 0.1% *BCR::ABL* ^IS^) and deep molecular response (DMR,MR4.5 ≤ 0.0032% *BCR::ABL* ^IS^). However, the EUTOS and ELTS were established after the introduction of imatinib and they cannot effectively identify patients with poor efficacy of imatinib as a first-line treatment.

Therefore, efforts have been made to explore new risk stratification methods to meet the needs of clinical practice. In order to select patients with first-line imatinib treatment failure (IMTF), Zhang Xiaoshuai et al. first proposed a predictive model for IMTF. The model consists of analyzing WBC count, hemoglobin concentration, blood basophil count, and ELTS score, which clinicians can use to estimate the probability of imatinib treatment failure (16). However, the model was established based on clinical data from a single center in Beijing, and there has been no relevant research in other regions of China. In addition, when clinicians treat patients with a warning of efficacy, they also recommend the replacement with 2^nd^ generation TKIs. Therefore, with the development of a new model, we aimed to effectively screen patients with poor efficacy of first-line imatinib and provide guidance for clinicians for the selection of first-line therapy.

Most researchers investigated that the achievement of MMR *(BCR::ABL* ^IS^ ≤ 0.1%) at 12 months is associated with a very low probability of subsequent loss of response and a high likelihood of achieving a subsequent DMR (MR4.0, *BCR::ABL* ^IS^ ≤ 0.01%), which may facilitate discontinuation of TKI therapy. Simultaneously, the significance of achieving MMR within 12 months has been established in various studies to correlate with event free survival (EFS) (17). This hallmark is recognized by the European LeukemiaNet as an optimal response and the National Comprehensive Cancer Network considers IS *BCR::ABL^IS^* ≥ 0.1% as an indicator for possible changes in therapy (18).

Hence, we defined patients with CML-CP who did not achieve MMR after 12 months of imatinib treatment as having poor efficacy. We explored the factors influencing the poor efficacy of imatinib as a first-line treatment in patients with CML-CP at 12 months by integrating sociodemographic and laboratory tests. Our study offers a simple but a novel tool to predict imatinib efficacy at 12 months in patients, which can be used for better stratification based on current risk classifications and as a guidance for clinicians for the selection of first-line TKI.

## 2 Methods

### 2.1 Cohort selection

We retrospectively enrolled 156 consecutive patients newly diagnosed with chronic-phase CML at our institution between January 2012 and June 2022. All patients were aged at least 18 years, diagnosed with CML-CP, receiving imatinib, followed up for at least 12 months, and who underwent analysis of *BCR::ABL* transcript type at 12 months of imatinib treatment.

### 2.2 Data collection

The baseline characteristics, such as sociodemographic characteristics, clinical co-variates, and treatment information were collected. Sociodemographic data included sex, age, annual household income, marital status, level of education, household registration, and comorbidity. The clinical co-variates data included white blood cell (WBC, ×109/L), hemoglobin (HGB, g/L), platelet (PLT, ×109/L), red blood cell distribution width (RDW, %), lymphocyte (LYM, ×109/L), monocyte (MONO, ×109/L), basophils (BAS, %), eosinophil (EOS, %), lactate dehydrogenase (LDH, U/L), alkaline phosphatase (ALP,U/L), marrow blasts (%), spleen size (cm), and blood blasts (%). The treatment information included the analysis of *BCR::ABL* transcript type at 12 months of age with imatinib mesylate treatment.

### 2.3 Diagnosis, responses and outcomes

The diagnoses, therapy responses, and outcomes followed the ELN recommendations. Complete cytogenetic response (CCyR) is equivalent to a negative FISH test (+/− 2%) and *BCR::ABL*≤ 1%.MMR was defined as *BCR::ABL* ≤ 0.1%. DMR was defined as *BCR::ABL* ≤ 0.01%/0.0032%. A poor response was defined as an unachieved MMR after 12 months of imatinib mesylate treatment.

### 2.4 Statistical methods

We summarized the patients’ baseline characteristics using descriptive statistics. Before variable selection, we transformed the continuous variables into categorical variables according to whether they followed a normal distribution and then selected the median or mean as cutoff points. Missing data on candidate prognostic variables were handled using multivariate imputation by chained equation (MICE).

Categorical data were presented as numbers (percentages) and analyzed using the chi-square test or nonparametric test for comparisons. Covariates with *P* < 0.20 in the uni-variable analyses were included in the multivariate analyses and backward stepwise regression (*P*<0.10)was used to select variables for inclusion in the nomogram.

A total of 1000 bootstrap resamples and leave-one-out cross validation were used to validate the nomogram internally. We assessed the discriminative ability and predictive accuracy of the model using the time-dependent area under the curve (AUC) of the receiver operating characteristic (ROC) curve, calibration plot, and Hosmer-Lemeshow curve. To assess the improvement in prediction compared with the Sokal, Euro, EUTOS, and ELTS scores, we used integrated discrimination improvement (IDI),continuous net reclassification index (NRI), continuous NRI, and decision curve analysis (DCA), which suggested that the new model had an improvement in predictive capacity compared with the old model when they were greater than zero (19–22).

We divided the patients into low and high risk subgroups according to optimal cut points calculated by the ROC curve and constructed chronic myeloid leukemia imatinib poor treatment (CML-IMP) risk stratification. We verify the applicability of the five risk scoring systems in evaluating cumulative CCyR, MMR and DMR by chi-square test and Fisher test.

All statistical analyses were performed using SPSS 22.0 and R version 4.0.2.

## 3 Results

### 3.1 Patients’ characteristics

**Figure 1:**
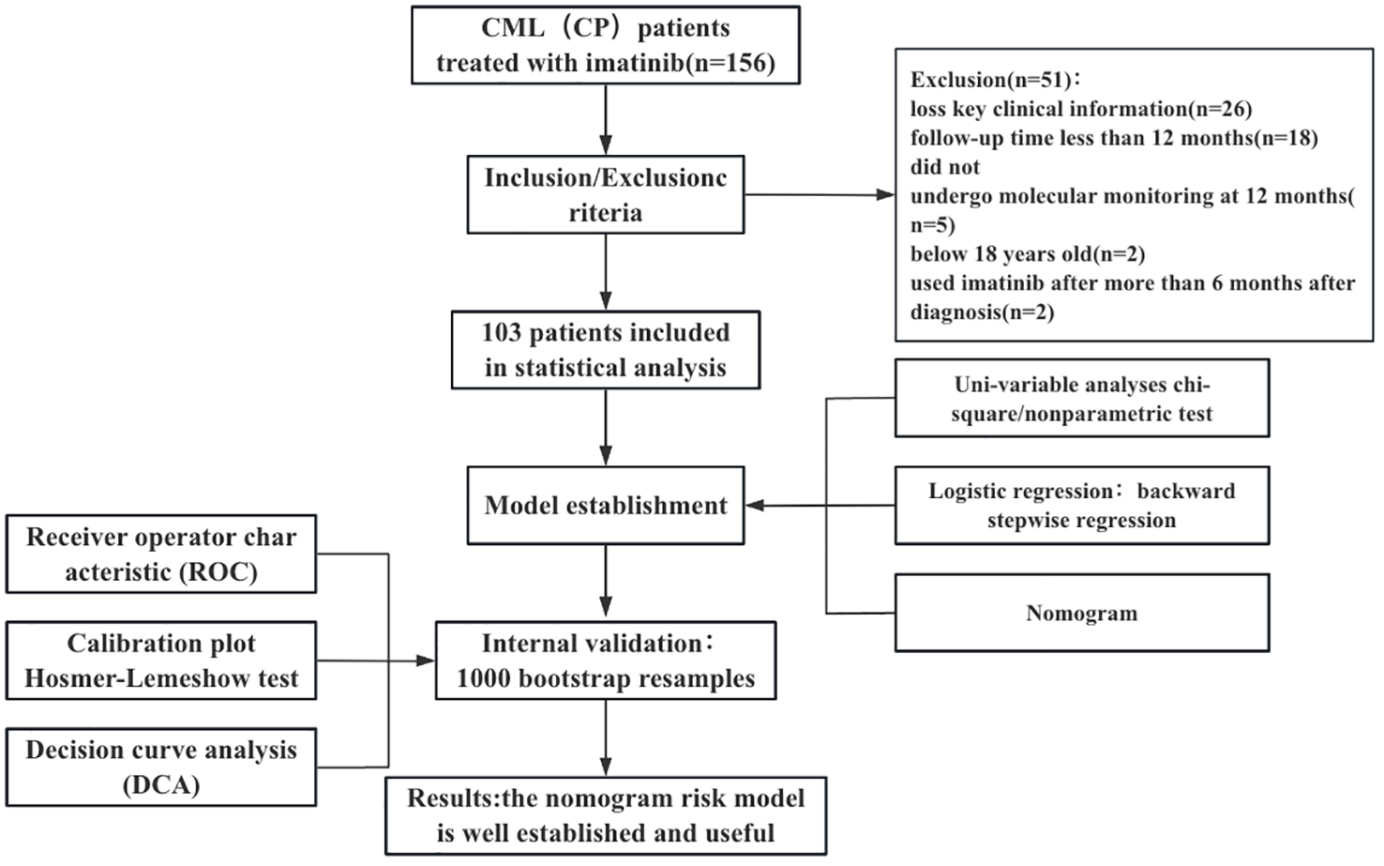
Flowchart of the research design.

We collected data from 156 patients who received imatinib mesylate initially. Out of the 156 patients, the following patients were excluded from the study: 26 patients with no available key clinical information; 18 patients with a follow-up period < 12 months; 2 patients treated with imatinib for more than 6 months after diagnosis; 5 patients who did not undergo molecular monitoring at 12 months of treatment with imatinib; and 2 patients below 18 years old. Finally, 103 patients were included in the study. We presented the baseline characteristics of the development cohort in the **Table 1**. The flowchart of the research design is shown in **Figure 1**.

**Table 1:** Baseline characteristics of development cohort.

Variables	Development(N=103)
Sex, n(%)	
Male	60(58.3)
Female	43(41.7)
Age(years), n(%)	
<60	71(68.9)
≥60	32(31.1)
Marital status, n(%)	
Married	87(84.5)
Single	12(11.7)
Divorced/Widowed	4 (3.9)
Household income(ten thousand yuan), n(%)	
<3	52(50.5)
3–8	31(31.0)
>8	20(19.4)
Level of education, n(%)	
Primary school and below	30(29.1)
Middle and high school	51(49.5)
University and above	22(21.4)
Household registration, n(%)	
Rural	54(56.5)
Urban	49(43.5)
Co-morbidity, n(%)	
No	72(75.8)
Yes	31(24.2)
WBC(×10^9^/L), n(%)	
< 100	42(40.8)
≥100	61(59.2)
HGB(g/L), n(%)	
<120	69(67.0)
≥120	34(33.0)
PLT(×10^9^/L), n(%)	
<450	72(69.9)
≥450	31(30.1)
RDW(%), n(%)	
<17	52(50.5)
>17	51(49.5)
LYM(×10^9^/L), n(%)	
< 7	48(46.6)
≥7	55(53.4)
MONO(×10^9^/L), n(%)	
< 3	45(43.7)
≥3	58(56.3)
BAS(%), n(%)	
< 4.7	49(47.6)
≥4.7	54(52.4)
EOS (%), n(%)	
< 1.5	50(48.5)
≥1.5	53(51.5)
Marrow blasts(%), n(%)	
< 2	46(44.7)
≥2	57(55.3)
LDH(IU/L), n(%)	
< 741	51(49.5)
≥ 741	52(50.5)
ALP(U/L), n(%)	
< 85	49(47.6)
≥ 85	54(52.4)
Spleen size (cm), n(%)	
< 4	53(51.5)
≥ 4	50(48.5)
Blood blasts(%), n(%)	
0	65(63.1)
≤ 2	30(29.1)
≥3	8(7.8)

WBC, white blood cell; HGB, hemoglobin; PLT, platelet; RDW, red blood cell distribution width; LYM, lymphocyte; MONO, monocyte; BAS, basophils; EOS, eosinophil; LDH, lactate dehydrogenase; ALP, alkaline phosphatase.

### 3.2 Development of a CML-IMP nomogram

**Figure 2:**
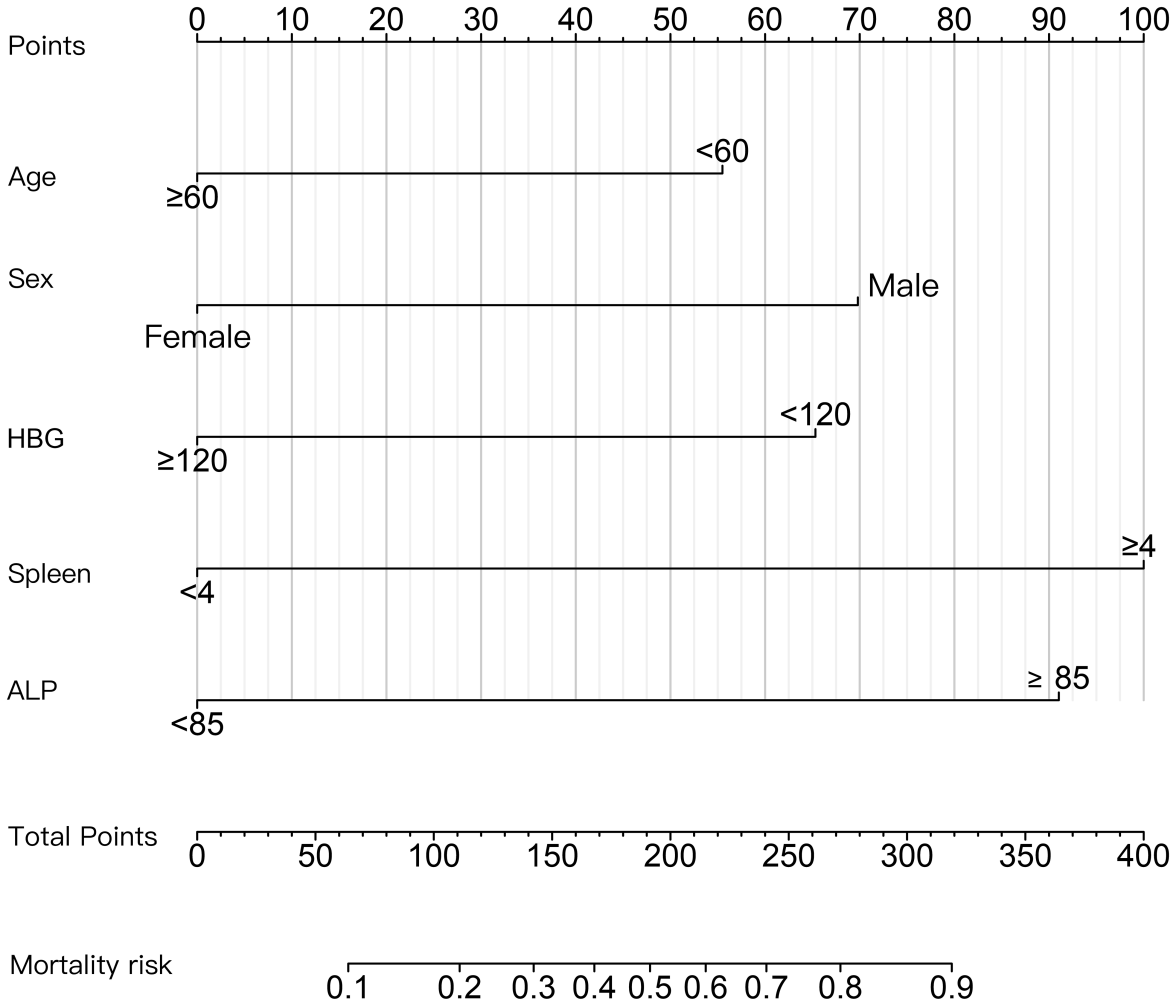
Nomogram for predicting the probability of poor efficacy of imatinib in the first-line treatment of CML-CP patients at 12 months. The value of each variable was scored on a point scale from 0 to 100, after which the scores for each variable were added together. That sum is located on the total points axis, which enables us to predict the probability of poor efficacy of imatinib.

The patients were divided into poor response cohort (N = 60) and optimal response cohort (N = 43), depending on whether they achieved MMR after 12 months of imatinib treatment. In uni-variable analyses, there were 13 variables with statistically significant differences between groups *(P* < 0.20): sex, age, annual household income, level of education, co-morbidity, white blood cell (WBC, ×109/L), hemoglobin (HGB, g/L), platelet (PLT,×109/L), red blood cell distribution width (RDW,%), monocyte (MONO, ×109/L), eosinophil (EOS,%), alkaline phosphatase (ALP,U/L),and spleen size (cm) (**Table 2**). We selected five variables to construct a new prognostic model in the form of a nomogram by using the multivariate logistic regression analysis (**Table 3**). The nomogram consisted of sex, age, hemoglobin levels, spleen size, and alkaline phosphatase levels and comprised a new prognostic model called the CML-IMP model for predicting the probability of poor efficacy of imatinib as a first-line treatment in patients with CML-CP at 12 months (**Figure 2**).

**Table 2:** Univariate logistic regression analyses in the development cohort.

Variables	Poor response cohort (N=60)	The optimal response cohort (N=43)	*P* values
Sex, n(%)			0.041
Male	40(66.7)	20(46.5)	
Female	20(33.3)	23(53.5)	
Age(years), n(%)			0.015
<60	47(78.3)	24(55.8)	
≥60	13(21.7)	19(44.2)	
Marital status, n(%)			0.615
Married	51(82.3)	38(88.4)	
Single	8(12.9)	4 (9.3)	
Divorced/Widowed	3 (4.8)	1 (2.3)	
Household income(ten thousand yuan), n(%)			0.105
<3	34(56.7)	18(41.9)	
3–8	17(28.3)	14(32.6)	
>8	9(15.0)	11(25.6)	
Level of education, n(%)			0.136
Primary school and below	20(33.3)	10(23.3)	
Middle and high school	30(50.0)	21(48.8)	
University and above	11(16.7)	12(27.9)	
Household registration, n(%)			0.309
Rural	34(56.7)	20(46.5)	
Urban	26(43.3)	23(53.5)	
Co-morbidity, n(%)			0.183
No	45(75.0)	27(62.8)	
Yes	15(25.0)	16(37.2)	
WBC(×10^9^/L), n(%)			0.009
< 100	18(30.0)	24(55.8)	
≥100	42(70.0)	19(44.2)	
HGB(g/L), n(%)			<0.001
<120	51(85.0)	18(41.9)	
≥120	9(15.0)	25(58.1)	
PLT(×10^9^/L), n(%)			0.183
<450	45(75.0)	27(62.8)	
≥450	15(25.0)	16(37.2)	
RDW(%), n(%)			0.012
<17	24(40.0)	28(65.1)	
>17	36(60.0)	15(34.9)	
LYM(×10^9^/L), n(%)			0.988
< 7	28(46.7)	20(46.5)	
≥7	32(53.3)	23(53.5)	
MONO(×10^9^/L), n(%)			0.036
< 3	21(35.0)	24(55.8)	
≥3	39(65.0)	19(44.2)	
BAS(%), n(%)			0.855
< 4.7	29(48.3)	20(46.5)	
≥4.7	31(51.7)	23(53.5)	
EOS (%), n(%)			0.040
< 1.5	24(40.0)	26(60.5)	
≥1.5	36(60.0)	17(39.5)	
Marrow blasts(%), n(%)			0.261
< 2	24(40.0)	22(51.2)	
≥2	36(60.0)	21(48.8)	
LDH(IU/L), n(%)			0.552
< 741	28(46.7)	23(53.5)	
≥ 741	33(53.3)	20(46.5)	
ALP(U/L), n(%)			0.003
< 85	21(35.0)	28(65.1)	
≥ 85	39(65.0)	15(34.9)	
Spleen size (cm), n(%)			<0.001
< 4	19 (31.7)	34(79.1)	
≥ 4	41(68.3)	9(20.9)	
Blood blasts(%), n(%)			0.220
0	37(61.7)	28(65.1)	
≤ 2	16(26.7)	14(32.6)	
≥3	7(11.7)	1(2.3)	

WBC, white blood cell; HGB, hemoglobin; PLT, platelet; RDW, red blood cell distribution width; LYM, lymphocyte; MONO, monocyte; BAS, basophils; EOS, eosinophil; LDH, lactate dehydrogenase; ALP, alkaline phosphatase.

**Table 3:** Multivariate logistic regression analyses in the development cohort.

Variables	Coefficient	Standard error	Wald value	*P* value	OR	95%CI
Sex	1.253	0.588	4.542	0.033	3.502	1.106~11.089
Age	-1.178	0.6	3.857	0.05	0.308	0.095~0.998
HGB	-1.671	0.754	4.912	0.027	0.188	0.043~0.824
Spleen size	2.398	0.755	10.094	0.001	10.996	2.506~48.261
ALP	1.644	0.599	7.521	0.006	5.175	1.598~16.754
Constant	-0.444	0.792	0.314	0.575	0.642	

HGB, hemoglobin; ALP, alkaline phosphatase.

### 3.3 Validation of the CML-IMP model

**Figure 3:**
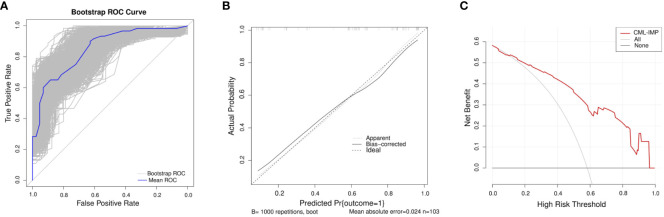
Evaluation of the CML-IMP model **(A)** Receiver operating characteristic curve for the CML-IMP model generated using bootstrap resampling (1000 times) **(B)** CML-IMP calibration plot. When the solid line (performance nomogram) was closer to the dotted line (ideal model), the prediction accuracy of the CML-IMP was better. **(C)** Decision curve analysis for the prediction model. The red solid line is for the prediction model, the gray line is for all patients with CML-CP, and the solid horizontal line indicates no patients have CML-CP. The graph depicts the expected net benefit per patient relative to the CML-IMP prediction of risk of poor efficacy of imatinib. The net benefit increases as the model curve is extended.

In the development of cohorts, 1,000 bootstrap resamples were used to internally validate the CML-IMP model. Leave-one-out cross validation indicated the CML-IMP model could classify 77.7% cases accurately. As for discrimination, time-dependent AUC was 0.851 (95%CI = 0.778~0.925) (**Figure 3A**). In addition, the results of the Hosmer–Lemeshow test showed that, χ2 = 5.492 (*P* = 0.704), proving that the model has good goodness of fit (**Figure 3B**). Moreover, the probabilities predicted by the nomogram matched well with the clinical outcomes (**Figure 3B**), and the decision curve showed that the model has potential clinical application value (**Figure 3C**).

### 3.4 Comparison of the CML-IMP models with standard prediction algorithms

**Figure 4:**
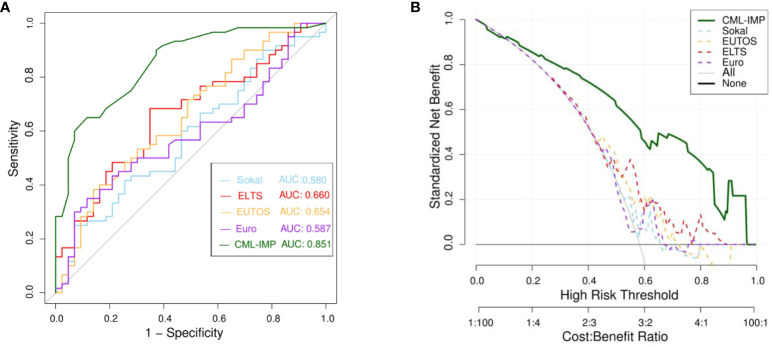
**(A)** Receiver operating characteristic curve (ROC) analyses for the prediction of probability of poor efficacy of imatinib of five models **(B)** The DCA was used to estimate clinical usefulness of the four models. The improvement in prediction precision of the CML-IMP model was compared to the Sokal, Euro, EUTOS, and ELTS.

To determine the most predictive model in clinical settings, we compared the CML-IMP model with the Sokal, Euro, EUTOS, and ELTS scores. **Figure 4A** showed that CML-IMP model improved the model accuracy such that the AUC compared with other models. We also assessed clinical effect by DCA which showed that the CML-IMP model could achieve positive net benefit over a wider range of risk threshold, with higher area under the decision curve analysis (AUDC) than Sokal, EUTOS, and ELTS score (**Figure 4B**). In addition, we evaluated the IDI, NRI, and continuous NRI to test the improvement in the prediction efficiency of the CML-IMP model and found that the new model improved the prediction of newly diagnosed CML-CP compared to the Sokal, Euro, EUTOS, and ELTS scores (**Table 4**).

**Table 4:** Comparing NRI, continuous NRI, and IDI with Sokal, Euro, EUTOS, and ELTS.

Index		Estimate	95% CI	*P* value
NRI	vs.Sokal	0.3903	0.1607~0.6199	<0.001
	Vs.Euro	0.3000	0.0477~0.5523	<0.050
	vs.EUTOS	0.2535	0.0327~0.4743	<0.050
	vs.ELTS	0.3066	0.0913~0.5219	<0.050
Continuous NRI	vs.Sokal	0.9225	0.5774~1.2675	<0.001
	Vs.Euro	0.9155	0.5663~1.2647	<0.001
	vs.EUTOS	1.0419	0.7059~1.3778	<0.001
	vs.ELTS	1.0085	0.6689~1.3482	<0.001
IDI	vs.Sokal	0.3775	0.2780~0.4769	<0.001
	Vs.Euro	0.3703	0.2721~0.4685	<0.001
	vs.EUTOS	0.3409	0.2512~0.4305	<0.001
	vs.ELTS	0.3055	0.2096~0.4014	<0.001

NRI, net reclassification index; IDI, integrated discrimination improvement.

Whats’more,we divided the development cohort into low and high risk subgroups according to optimal cut points calculated by the ROC curve.The low risk subgroup predicted the probability of poor outcome ≤0.6628,and the high subgroup predicted the probability of poor outcome >0.6628. There are 59 patients (57.3%)in low risk group and 44 patients(42.7%) in high risk group according to CML-IMP risk stratification. In predicting treatment response, only Sokal and CML-IMP scoring systems can effectively predict the cumulative acquisition rates of CCyR, MMR, and DMR (*P*<0.05) (**Table 5**).

**Table 5:** Evaluation of molecular response achievement in patients with CML-CP using five scoring systems.

Scoring systems	The rate of CCyR(%)	P	The rate of MMR (%)	P	The rate of DMR (%)	P
Sokal		0.004		0.004		0.031
Low risk	47(97.9)		40(83.3)		30(62.5)	
Intermediate risk	35(89.7)		31(79.5)		27(69.2)	
High risk	11(68.8)		7(43.8)		5(31.3)	
Euro		0.035		0.247		0.390
Low risk	39(41.9)		32(41.0)		25(40.3)	
Intermediate risk	50(53.8)		43(55.1)		35(56.5)	
High risk	4(66.7)		3(3.8)		2(33.3)	
EUTOS		0.363		0.352		0.281
Low risk	79(84.9)		67(85.9)		54(62.8)	
High risk	14(82.4)		11(64.7)		8(47.1)	
ELTS		0.166		0.200		0.077
Low risk	59(93.7)		51(81.0)		43(69.4)	
Intermediate risk	27(87.1)		22(71.0)		16(47.1)	
High risk	7(77.8)		5(55.6)		3(33.3)	
CML-IMP		0.017		0.020		<0.001
Low risk	57(61.3)		50(64.1)		45(72.6)	
High risk	36(38.7)		28(35.9)		17(38.6)	

CCyR, complete cytogenetic response; MMR, major molecular response; DMR, deep molecular response.

## 4 Discussion

In this retrospective study, we established and validated a CML-IMP model that included characteristics such as, age, sex, HBG, spleen size, and ALP. The novel CML-IMP model performed well in terms of discrimination, calibration, clinical usefulness, and improvement in prediction, suggesting a good prognostic value in predicting the probability of poor efficacy of imatinib as a first-line treatment strategy for patients with CML-CP at 12 months.

Current models such as Sokal, Euro, EUTOS, and ELTS scores predict TKI treatment response and outcomes based on the clinical characteristics of patients, WBC count at initial diagnosis, HGB, comorbidities, *BCR::ABL* transcript types, and high-risk additional chromosomal abnormalities. Previous studies have confirmed that sociodemographic factors are significantly associated with the prognosis of patients with chronic myeloid leukemia (23–28). Therefore, in this model, sociodemographic factors were considered, including patient age, sex, annual household income, marital status, level of education, household registration, and other factors. According to research reports, the OS rate of patients with CML in the United States is significantly lower in low-income populations, males, and unmarried patients (23). Similarly, in our study, sex, age, annual household income, and level of education were found to be significant in the univariate analysis; however, in the multivariate analysis, annual household income and level of education were not included, which may be related to our small sample size and confounding factors.

We retrospectively enrolled 156 patients newly diagnosed with CML-CP. The median age of patients with CML-CP at diagnosis was 53 years (18–77). Patients with CML-CP included 60 males and 43 females. Our analysis showed that it is easier to achieve an optimal response (MMR) after 12 months of imatinib treatment in elderly patients (29). In China, a study based on a large population found that with an increase in age, high WBC levels, low hemoglobin concentrations, high percentage of blood basophils decreases at the time of initial diagnosis, indicating that the degree of proliferation and invasion of the disease is gradually decreasing, which also explains why it is easier for elderly patients to achieve MMR when treated with imatinib for 12 months. Contrarily, increased age is a risk factor in the Sokal and ELTS scores, but this may be due to different endpoints of the scores; Sokal score considers the patient’s OS as the study endpoint whereas the ELTS score considers CML-related death as the study endpoint. It has been suggested that the incidence of serious hematological and non-hematological adverse reactions in elderly patients increased, resulting in a significant increase in the proportion of patients who have reduced, interrupted, or discontinued TKI treatment. In addition, there were more comorbidities in the elderly, which may explain the difference in the results between the two studies. Moreover, many real-world retrospective cohort studies have identified poor prognostic factors in male patients with CML, consistent with our findings (23, 30).

Previous studies have proposed factors potentially affecting the prognosis of patients with CML-CP, such as high WBC count, low hemoglobin concentration, high percentage of blood basophils, high ELTS risk score, high-risk ACAs, and possibly some *BCR::ABL* transcript types (31–33). However, these prognostic factors are controversial and have low prediction accuracy, whereas our new model chose several factors with high prediction discrimination, calibration, and clinical applicability. Additionally, ALP levels were independently associated with imatinib efficacy in our study. ALP hydrolyzes phosphate esters to produce phosphoric acid in an alkaline environment and is a hallmark enzyme after neutrophil mitosis. It has been reported that there is a certain relationship between the ALP level in patients with CML and the changes in the patient’s condition and clinical stage (34).

Furthermore, the CML-IMP model has a greater ability to recognize the risks affecting the efficacy of imatinib as a first-line treatment strategy in patients with CML-CP than the conventional staging system. The nomogram demonstrated its potential value in clinical practice. It can help clinicians select patients who need 2^nd^ generation TKI treatment during initial therapy.

Although the CML-IMP model performed well, the present study has some limitations. First, this was a single-center study of Chinese patients from a single region, which may have limited its generalization. Although our nomogram was validated using bootstraps with 1000 resamples, future prospective multicenter studies are still needed for external validation. Secondly, sociodemographic factors such as marital status, annual household income, educational level, etc., may change after the diagnosis of the disease. Thirdly, we excluded patients who were not regularly followed up at the time of study inclusion, which may lead to selection bias. Finally, this is a real-world retrospective cohort study that inevitably shows some missing values, but we processed the missing data by using multiple imputation methods to minimize the impact of data deficiencies. Therefore, prospective studies are needed in the future to reduce the impact of selection bias and data loss on the conclusions, and further improve the predictive ability of the model.

## 5 Conclusions

In summary, we developed a user-friendly nomogram with increased accuracy, good clinical utility, and more precise prognostic prediction than the conventional staging system, which could potentially predict the probability of poor efficacy of imatinib mesylate in the first-line treatment of patients with CML-CP at 12 months. It can help clinicians accurately select patients who need 2^nd^ generation TKI treatment as the initial therapy in clinical practice and complement the current risk stratification of patients with CML-CP.

## Data availability statement

The raw data supporting the conclusions of this article will be made available by the authors, without undue reservation.

## Ethics statement

The studies involving humans were approved by the Ethics Committee of the Second Affiliated Hospital of Xi’an Jiaotong University. The studies were conducted in accordance with the local legislation and institutional requirements. The participants provided their written informed consent to participate in this study.

## Author contributions

YXL: Project administration, Funding acquisition, Formal analysis, Visualization, Resources, Conceptualization, Supervision, Investigation, Writing – review & editing, Validation, Software, Methodology, Data curation, Writing – original draft. YLZ: Software, Visualization, Writing – original draft, Data curation, Writing – review & editing. JW: Writing – review & editing, Methodology, Data curation. ALH: Writing – review & editing, Methodology, Data curation. WGZ: Data curation, Writing – review & editing, Methodology. XMC: Methodology, Data curation, Writing – review & editing. YXC: Writing – review & editing, Methodology, Data curation. JL: Data curation, Methodology, Writing – review & editing. PYZ: Writing – review & editing, Methodology, Data curation. JLW: Data curation, Writing – review & editing, Methodology. WHZ: Writing – review & editing, Methodology, Data curation. YY: Writing – review & editing, Methodology, Data curation. XM: Software, Resources, Writing – review & editing. SPC: Resources, Writing – review & editing, Software. LJZ: Resources, Software, Writing – review & editing. TW: Software, Writing – review & editing, Resources. XGW: Resources, Writing – review & editing, Software. XRM: Supervision, Writing – original draft, Investigation, Writing – review & editing, Visualization, Methodology, Formal analysis, Data curation.
